# Review: Mitochondrial Defects in Breast Cancer

**DOI:** 10.4137/cmo.s524

**Published:** 2008-04-01

**Authors:** Josefa Salgado, Beatriz Honorato, Jesús García-Foncillas

**Affiliations:** 1 Clinical Genetics Unit, University Clinic of Navarra (CUN), Avda, Pio XII, 36, 31008 Pamplona, Navarra, Spain; 2 Laboratory of Pharmacogenomics, Centre for Applied Medical Research (CIMA), Avda, Pio XII 55, 31008 Pamplona, Navarra, Spain

**Keywords:** mitochondrial DNA, breast cancer, mutations, heteroplasmy

## Abstract

Mitochondria play important roles in cellular energy metabolism, free radical generation, and apoptosis. Mitochondrial DNA has been proposed to be involved in carcinogenesis because of its high susceptibility to mutations and limited repair mechanisms in comparison to nuclear DNA. Breast cancer is the most frequent cancer type among women in the world and, although exhaustive research has been done on nuclear DNA changes, several studies describe a variety of mitochondrial DNA alterations present in breast cancer. In this review article, we to provide a summary of the mitochondrial genomic alterations reported in breast cancer and their functional consequences.

## Introduction

**Figure 1:**
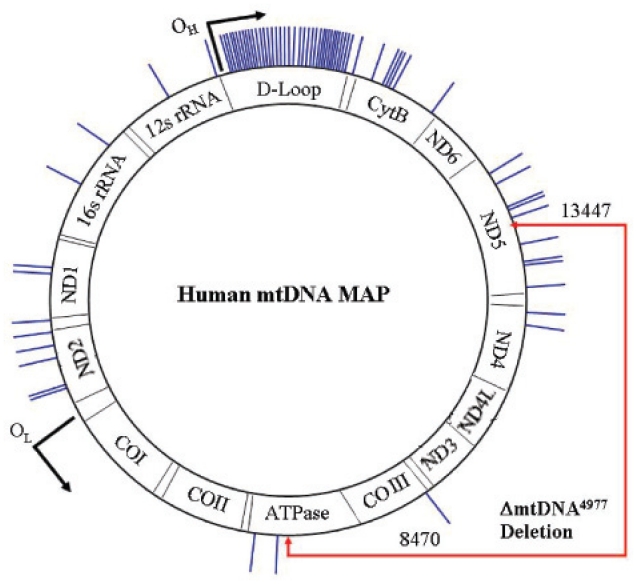
mtDNA point mutations in breast cancer described in the literature. Every tick represents a mutation locus detected. **Abbreviations:** 12S: 12S ribosomal RNA; 16S: 16S ribosomal RNA; ND1, 2, 4, 5 and 6: NADH dehydrogenase subunit 1, 2, 4, 5 and 6; ATPase: ATP Synthase F0 subunit 8; COI, II and III: cytochrome *c* oxidase subunit I, II and III; Cyt B: cytochrome *b*; O_H_: H-strand replication origin; O_L_: L-strand replication origin.

Mitochondria are cytoplasmic semi-autonomously functioning organelles, which contain a resident genome, and replicate, translate and transcribe their own DNA (mtDNA) (Anderson et al. 1981; Taanman, 1999). Each organelle contains 2–10 copies of mtDNA molecules, and each human cell contains thousands of copies which varies in number with the cell type (Chen and Butow, 2005). Mitochondria are responsible for generating approximately 90% of cellular ATP through the process of oxidative phosphorylation (OXPHOS) and play essential roles in cellular energy metabolism, free radical generation, and programmed cell death (Chan, 2006). Human mtDNA is a circular double-stranded molecule consisting of 16.571bp encoding 13 polypeptides that constitute respiratory chain complexes, as well as 2 rRNAs and a set of 22 tRNAs for mitochondrial protein synthesis (Anderson et al. 1981; Andrews et al. 1999). The mtDNA undergoes replication using a different origin for each of the two strands, the purine (guanine) abundant H-strand, and the pyrimidine (cytosine) abundant L-strand. The displacement (D)-loop encompasses 1124 bp, between nt 16024 and nt576, and is the only major non-coding region of the mitochondrial genome. It contains the origin of replication for H-strand synthesis, both mitochondrial transcription promoters (Lutz et al. 1997; Taanman, 1999), and has a triple-stranded DNA structure that is more sensitive to oxidative damage due to the exposure of the displaced single strand in the arrested replication intermediate (Clayton, 2000). It is also the attachment point of the mtDNA molecule to the mitochondrial inner membrane (Clayton, 1992). Replication is initiated at the H-strand origin, and proceeds to two-thirds around the genome until the L-strand origin is exposed, thus initiating L-strand synthesis (Fig. 1). Unlike nuclear DNA (nDNA), mtDNA may replicate more than once during each cell cycle, or not at all. Moreover, mtDNA may replicate in non-dividing cells. Compared with nDNA, mtDNA is more vulnerable to damages due to the lack of protective histones, limited DNA repair capacity, lack of introns, and is exposed continuously to endogenous reactive oxygen species (ROS) (Chen and Butow, 2005; Higuchi, 2007). Since all of the genes in mtDNA are crucial for biogenesis and bioenergetics function of mitochondria, any mtDNA alteration may result in impairment of OXPHOS and enhanced ROS production, which in turn, may accelerate the rate of mtDNA mutations (Higuchi, 2007; Lee and Wei, 2005). Mutated mtDNA and wild-type mtDNA molecules can coexist in the same cell, tissue or organ in a state called heteroplasmy. Mitochondria containing only mutated DNA (or only wild-type mtDNA) are called homoplasmic. The study of genotype-phenotype relationships in mitochondrial diseases has shown clearly the existence of a “phenotypic threshold effect”, which can be characterized by the following: (i) a low proportion of wild-type mtDNA coexisting with mutated mtDNA allows a normal phenotype to be maintained, but (ii) a small decrease in this proportion below a threshold value alters the phenotype.

## Mitochondrial DNA Mutations and Breast Cancer

Mitochondrial defects have long been suspected to play an important role in the development and progression of cancer. Over 70 years ago, Warburg pioneered the research on mitochondrial respiration alterations in the context of cancer and proposed a mechanism to explain how they evolve during the carcinogenic process. He hypothesized that a key event in carcinogenesis involved an “injury” to the respiratory machinery, resulting in compensatory increases in glycolytic ATP production. Due to the inherent inefficiency of glycolytic ATP generation, this represents a unique metabolic state of the malignant cells and would require high consumption of glucose to fulfil cellular energy requirements. Therefore, cancer cells actively metabolize glucose and produce excessive lactic acid while, at the same time, consume oxygen via OXPHOS. Warburg called this phenomenon “aerobic glycosysis” (Warburg, 1930; Warburg, 1956). Wargburg’s observation stimulated many investigators to analyse mitochondrial function in tumor cells. Since then, many studies have reported mtDNA abnormalities in colorectal, bladder, head and neck, lung, pancreatic, gastric, hepatic, renal, ovarian and breast cancers and haematological diseases among others (Brandon et al. 2006; Chatterjee et al. 2006). The role of mtDNA mutations in tumor development is still unclear and only a subset of the mutations observed are predicted to have a relevant effect. The mtDNA mutations could arise either in the germ line and predispose to cancer (oncogene germline mutations), or in the mtDNA of the tissues (tumor-specific somatic mutations) and participate in the tumor progression process. Breast cancer is the most commonly diagnosed cancer of women and the second leading cause of cancer deaths among women. On one hand, inherited mutations in the breast cancer susceptibility genes, BRCA1 and BRCA2, predispose to breast, ovarian, and other types of cancer (Thompson and Easton, 2004). On the other hand, according to epidemiological studies, prolonged exposure to estrogen, including an earlier menarche, and prolonged reproductive stimulation during lifetime, significantly associate with an increased risk of sporadic breast cancer (Oldenburg et al. 2007). Until recently, research has focused on nDNA changes, while alterations in mtDNA were largely ignored. Nowadays, there has become increasing awareness that mtDNA mutations might be more worthy of the kind of scrutiny once applied only to nDNA. Several studies indicate that most breast cancers harboured mtDNA that contains a variety of alterations including point mutations (germline and/or somatic), mtDNA depletion and microsatellite instability (MSI).

### Germline mtDNA mutations in breast cancer

Several mtDNA population polymorphisms have been associated with increased breast cancer risk, stressing the importance of germline mtDNA mutations in the aetiology of certain cancers. One of those mutations is the germline T16189C substitution in one of the poly (C)-T-poly(C) structural motifs within the D-loop region. The T to C change in the wild-type (C_5_TC_4_) generates a long run of C residues considered to be a source of enhanced instability. Some authors have associated this variant with susceptibility to endometrial cancer (Liu et al. 2003) and breast cancer (Wang et al. 2006). It has been proposed as a potential marker for cancers with metabolic risk factor, since is has been associated significantly with type II diabetes (Poulton et al. 2002) and it has long been known that diabetes mellitus is an important epidemiologic risk factor for endometrial and breast cancers (Weiderpass et al. 1997). Another intensively studied change is a G10398A polymorphism (codon 114) which results in a non-conservative amino acid substitution of threonine (encoded by the A allele) for alanine (encoded by the G allele), within the NADH Dehydrogenase (ND3) subunit of Complex I. The 10398A allele was linked to increased risk for invasive breast cancer in both pre- and post-menopausal African-American women, although the mechanism underlying this relationship remains to be elucidated (Canter et al. 2005). Since Complex I is a key component of the mitochondrial electron transport chain, the amino acid change may increase the rate of electron leakage and ROS generation, contributing to mtDNA and nDNA mutations and cumulative mitochondrial dysfunction which could contribute to disease. Many factors, other than the mtDNA G10398A polymorphism are likely to be important in the development and progression of invasive breast cancer in African-American women (Hall et al. 2005). In a very recent study Bai et al. showed that 10398A > G, 9055G > A and 16519T > C may increase the risk of developing breast cancer or are in linkage disequilibrium with functional variants that increase this risk. However, 3197T > C and 13708G > A have a protective effect or are in linkage disequilibrium with protective variants. In addition, this group studied mtDNA haplogroups and find an important role in disease expression. Clusters of mtDNA polymorphisms constitute haplogroups that have been extensively used to track human migrations and to explore variation in disease phenotypes. Within the 10 European haplogroups observed there was much higher proportion of breast cancer cases compared with controls in haplogroup K (defined by 29 polymorphic sites), whereas opposite result is observed for haplogroup U. Therefore, mitochondrial genetic background would play a very important role in modifying the individual’s risk to breast cancer (Bai et al. 2007).

### Somatic mtDNA mutations in breast cancer

While interpretation of linking germline mtDNA mutations to cancer can be controversial due to the high background frequency of functional mtDNA polymorphisms, studies of somatic mtDNA mutations could be easy to handle since the cancer cell should have the neoplastic mtDNA mutation while the normal tissue should not. Parrella et al. determined the frequency and distribution of mtDNA mutations in 18 primary breast tumors by direct sequencing. 42% were deletions or insertions within the D-loop, 58% were single-base substitutions in the coding (ND1, ND4, ND5 and cytochrome b genes) or non-coding regions (D-loop). In 25% of the cases the mutations detected in the coding region led to amino acid substitutions in the protein sequence. Identical changes were found in breast nipple fine-needle aspirate fluid (FNA) and metastatic positive lymph nodes (Parrella et al. 2001). Zhu et al. described 45 somatic mutations in breast cancer tissue and in nipple aspirate fluid, 35 of those were at unique loci. Mutations at nine of the loci (seven in the D-loop and two in non-D loop areas) have been reported in other cancer types suggesting that these sites were susceptible to mutation in a variety of human malignancies (Bianchi et al. 1995; Polyak et al. 1998; Fliss et al. 2000; Parrella et al. 2001). At the same time, and although not all the FNA had sufficient tumor cells to detect the mutations identified in the tissue, FNA represent a promising non-invasive approach to obtain breast epithelial cells (Zhu et al. 2005). Tan et al. used a combination of temporal temperature gel electrophoresis and direct DNA sequencing to screen the complete mitochondrial genome for mutations in 19 sets of paired normal and breast tumor tissues. Somatic mutations were identified in 74% of the patients. The bulk of the mutations (82%) were restricted to the D-loop region reinforcing, again, the findings that this region is a mutation hot spot (Tan et al. 2002). D-loop alterations may interfere with the sequence in the promoter areas and modify the affinity to the inducers or modulators of mtDNA transcription, thus putatively resulting in changes in the rate of transcription, RNA primer synthesis, and mtDNA replication (Clayton, 2000; Shadel and Clayton, 1997). Indeed, mtDNA content of hepatocellular carcinoma and breast tumors was significantly decreased in 71% and 88% of patients with D-loop region mutations (Lee et al. 2004; Yu et al. 2007). Data suggest a possible contribution of mtDNA D-loop mutation to alterations in mtDNA level. A representation of mtDNA point mutations in breast cancer, described in the literature, is shown in Figure 1. The most frequently observed mtDNA mutation in human tissues is the ΔmtDNA^4977^ deletion. This mutation affects genes encoding 7 polypeptide components of the mitochondrial respiratory chain, and 5 of the 22 tRNAs necessary for mitochondrial protein synthesis (Porteous et al. 1998). The ΔmtDNA^4977^ mutation was initially reported as occurring in one out of seven breast cancer samples (Bianchi et al. 1995). Further studies reported a different pattern of ΔmtDNA^4977^ mutation in tumors compared to normal tissue (Dani et al. 2004; Tseng et al. 2006). Recently, high sensitivity and specificity assays show that this ΔmtDNA^4977^ mutation is ubiquitous in both tumor and non-tumor tissues of both breast cancer and benign breast disease (Ye et al. 2007). Bibliographic data do not support the notion that the ΔmtDNA^4977^ mutation plays a major role in breast carcinogenesis (Schwartz et al. 2006; Tseng et al. 2006; Zhu et al. 2004). The amount of ΔmtDNA^4977^ mutation that accumulates may not be sufficient, by itself, to cause a significant defect or dysfunction in breast cancer cells (Ye et al. 2007). Other large deletions (ΔmtDNA^3938^, ΔmtDNA^4388^ and ΔmtDNA^4576^) require further investigations. The first two have been found to be more frequent in cancerous than in adjacent histologically normal breast specimens, but were also identified in normal tissue from women without breast cancer. The last one, was detected in 13% of histologically normal specimens from a breast with cancer but in 77% of cancer specimens from the same breast (Zhu et al. 2004).

### Somatic mtDNA mutations could not be what they look like

To learn more about the nature of somatic mtDNA mutations in tumors, Brandon et al. recruited all data on somatic mtDNA, found in different types of cancers, and compared them with the mtDNA variant sites contained in their global human mtDNA database of 1920 complete mtDNA sequences plus 532 coding region mtDNA sequences (Brandon et al. 2006; Brandon et al. 2005). Surprisingly, 72% of the somatic mtDNA mutations were also sequence variants in population specific haplogroups database. A clear example is the nt13708 G to A missense mutation in ND5 (A458T), reported in breast cancer tumor (Parrella et al. 2001). In African L0-L3, Asian M, and at the base of Eurasian N mtDNAs, the nt13708 is a G (458A). However, at the root of European haplogroup J, and in important sublineages of the European X and Asian B haplogroups, the nt13708 is an A (458T). Therefore, the tumor-specific mutation results in the conversion of the mtDNA sequence to a Eurasian-like allele. Authors suggest that cancer cells acquire some of the same functional mtDNA mutations that confer adaptive advantages in population haplotypes, as cells migrate into new environments. They consider two main classes of mtDNA mutations: *tumorigenic mutations* and *adaptive mutations*. The first ones are found only in tumors and lead to a relatively severer deleterious effects on mitochondrial metabolism and ROS production. The second ones, alter the same nucleotides as population variants and may have a milder impact on mitochondrial OXPHOS, and help the tumors to adapt to adverse environments. Moreover, these two different classes of somatic mutations might arise at different times, being tumorigenic mutations with advantage in the initial phases of tumor growth. In this early stage, tumor is hypoxic and thus can tolerate OXPHOS deficiency since its generating ATP by glycolysis. By contrast, adaptive mtDNA mutations may be more advantageous when the tumor becomes vascularized and/or invade new environments as it metastasizes (Brandon et al. 2006). Therefore, understanding mtDNA variations in cancer cells provide new major insights into the aetiology of solid tumors.

## Mitochondrial DNA Copy Number in Breast Cancer

The evidence regarding the role of mtDNA content in mammary tumorigenesis, and its relationship with clinicopathological factors and patient outcome, is limited. It has been shown that a reduction of mtDNA content occurred in breast tumors, but this proportion was greater for papillary thyroid carcinomas, suggesting that changes in mtDNA content during carcinogenesis may be regulated in a tumor-specific manner (Mambo et al. 2005). In a study of 60 pairs of tumors, and corresponding no tumorous breast tissues, results indicated that 63% of the tumors had a significant lower mtDNA content compared to their corresponding non-tumorous breast tissue (Tseng et al. 2006). The analysis of 59 pairs of breast tumors and adjacent non-tumorous tissues has shown recently that 78% of the cases carried a reduction of mtDNA content compared to the corresponding non-tumorous cells. It has been well documented that ER-negative breast tumors have significantly more aggressive clinical behaviour and are unresponsive to anti-estrogenic therapy ((EBCTCG). 2005). A reduced mtDNA copy number tends to have a significant correlation with ER status (Yu et al. 2007). Several lines of evidence have demonstrated that altered mtDNA content may lead to a functional defect in oxidative phosphorylation and affect the respiratory activity of the cells, ultimately changing their growth behaviour (Isidoro et al. 2004). The mtDNA level reduction can be due to: (i) mtDNA D-loop mutations and (ii) dysfunction of p53 gene, among other causes. In the first case, and as it has been mentioned before, there is a possible contribution of mtDNA D-loop mutations to alterations in mtDNA level. In the second case, it has been reported that the p53 plays an important role in maintaining mitochondrial genetic stability via the interaction with mtDNA polymerase γ(Achanta et al. 2005; Heyne et al. 2004). Loss of p53 could lead to increased mtDNA vulnerability to exogenous and endogenous damage, subsequently elevating frequency of mtDNA mutations (as for example D-loop mutations), and therefore mtDNA depletion.

## Mitochondrial DNA and Microsatellite Instability (MSI) in Breast Cancer

Microsatellites are short tandem repeats of sequence motifs, usually ranging from one to five DNA bases. MSI is the change of length in any such stretch, due to either insertions or deletions of repeating units in a microsatellite, within tumor DNA compared to that of normal tissue. Nuclear MSI is thought to result from a defect in the mismatch repair system, which leads to failure in correction of the slippage errors made by DNA polymerases during replication. Nuclear MSI was first described in colorectal cancer (Thibodeau et al. 1993) and subsequently observed in most cancers (Lawes et al. 2003). However, instability in these terms of the mitochondrial genome, which is also subject to genetic alterations in cancer cells, (Bianchi et al. 2001; Richard et al. 2000) is worse characterized, and the responsible mechanisms of generation are still unclear. Wang et al. studied 12 candidate microsatellite regions within the entire mtDNA genome of a series of primary cervical (71), endometrial (62), ovarian (73) and breast cancer (51) patients. One relevant finding of this study was that the poly (C)-T-poly(C) structural motifs in the D-loop seem to be prone to MSI (Wang et al. 2006). The wild-type sequences (according to revised Cambridge sequence, http://www.mitomap.org/) of these markers are C_7_TC_5_ at nt303, C_5_TC_4_ at nt956 and C_5_TC_4_ at nt16184 and their instability patterns are different. At nt303, alterations were detected upstream to the T residue, the mtMSI manifestation being (C)_7–11_ TC_5_. At nt16184, mtMSI presents a particular pattern, consequential to a T to C germline substitution at nt16189, which has been discussed before. The nt956 showed both types of mtMSI. Authors found that mtMSI had different profiles and frequency in the four types of cancer, suggesting that different tumors may have generated different extents of mtMSI during carcinogenesis (Wang et al. 2006). On the contrary, no microsatellites in the coding region containing poly (A), poly (T) and poly (C) were unstable. It has been suggested that polyC-instability might be related to a highly mitogenic history and to progressive tumors, in particular, tumors that had already metastasized at the time of diagnosis. Nevertheless, mutations in the (C)n non-coding repeats could not be selected during tumor progression, but associated with higher replication rates of mtDNA in tumors because of their inherent mutability (Schwartz et al. 2006). Although mtMSI hot spots have been identified, it remains a challenge to address their functional impact.

## Homoplasmy/Heteroplasmy

**Figure 2:**
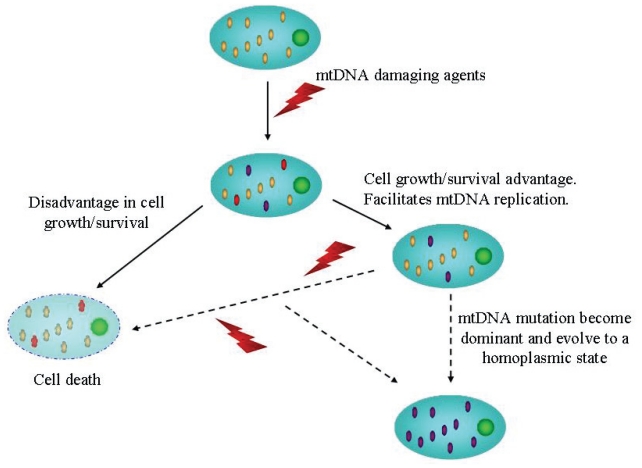
The *classic selection* method used to explain the expansion of mtDNA homoplasmy in tumors (in the *random genetic drift,* human tumors are expected to contain homoplasmic mitochondrial mutations based only on random mutagenesis and segregation of mtDNA).

Published data show that the majority of somatic mtDNA mutations are homoplasmic, indicating that mutant mtDNA had become dominant in the cancer cells, although the mechanism(s) for the development of mtDNA homoplasmy in cancer tissue is controversial. The lack of histones protection, limited repair ability, and close proximity to the electron transport chain make mtDNA far more susceptible to DNA damaging agents (ROS and/or certain anticancer drugs and radiation). Since such induced mutations do not involve all copies of mtDNA, an initial mutation is heteroplasmic with the mutated mtDNA being the minority. Two models, *classic selection* and *random genetic drift*, have been developed to explain the expansion of homoplasmy in tumors (Cavelier et al. 2000; Coller et al. 2001). The first interpretation is that expansion of the mutation was driven by strong selection for the mutant phenotype (Fig. 2). In this context, if a mutation leads to a disadvantage in cell growth or survival, such mutation is deemed to diminish. Conversely, if a mutation confers cell growth/survival advantage or facilitates mtDNA replication, such a mutation is likely to survive the selection and emerge as a heteroplasmic mutation. Depending on the degree of growth/survival advantage, cells carrying the mtDNA mutation may eventually become dominant and evolve to establish a homoplasmic mutant state. It is possible that mtDNA mutations cause a functional change that compromises the efficiency of the electron transport chain, leading to electron flow bifurcation and increased generation of ROS. A moderate increase of ROS has been found to stimulate cellular proliferation and mitochondrial biogenesis (Liu et al. 2002). The increase in ROS production may cause further damage to both mtDNA and nDNA, leading to cancer development, genetic instability, and disease progression. The second model, published by Coller et al. conclude that human tumors are expected to contain homoplasmic mitochondrial mutations based only on random mutagenesis and segregation of mtDNA (Coller et al. 2001). By using extensive computer modelling programs, authors show that when the mtDNA mutation occurs in a tumor progenitor cell, homoplasmy can be archived entirely by chance through unbiased mtDNA replication and sorting during cell division, without selection for physiological advantage. They estimate the mtDNA mutation rate as 7–10 mutations per base pair per generation, and the number of generations of cell growth experienced by a carcinoma founder cell as 600. Model predicts that 58% of tumors contain at least one homoplasmic point mutation. This fraction is close to the experimental observation that 48% of tumors from which essentially the entire mitochondrial genome was sequenced, contain at least one homoplasmic mtDNA mutation (Fliss et al. 2000; Polyak et al. 1998). Moreover, this model also correctly predicts the distribution of the number of mutations per tumor, and it is relatively stable with respect to changes in parameters such as the number of mitochondria per cell, the mutation rate and the number of generations. Therefore it could be argued that, some mitochondrial mutations may provide a selective advantage for the carrier mtDNA copy of the host cell. However, although selection may occur in some instances, it is not necessarily the only cause. A large fraction of human tumors are expected to be homoplasmic for mtDNA point mutations based solely on reported mitochondrial mutant fractions and cell growth kinetics of tumors. Whereas it cannot be excluded the possibility that selection occurs in a subset of tumors, physiological advantage or a role for mitochondrial mutations in tumorigenesis, may not be the only cause to explain the existence of homoplasmy or its observed frequency. Finally, although the mitochondrial and nuclear genomes are physically distinct, there is constant communication between the two genomes to carry out many of the mitochondrial functions. The identification of a large number of genes whose expression is influenced by mitochondrial function provides pathways affected by the impairment of mitochondria in pathological conditions (Delsite et al. 2002). And this impairment would be, at the same time, related to the grade of mitochondrial heteroplasmy. In this context we have to consider that phenotypic manifestations of mitochondrial defects occur when a threshold level is exceeded. Depending on the gene affected by the mutation in mtDNA, and the grade of heteroplasmy, complementation can occur at different levels: transcription, translation, enzyme assembly and activity, mitochondrial ATP synthesis, cell activity and, finally, organ function (Rossignol et al. 2003).

## Summary

The presence of mtDNA mutations in cancer is consistent with the intrinsic susceptibility of mtDNA to damage and constitutive oxidative stress. The central role of the mitochondria in energy production, ROS generation and apoptosis regulation, combined with a variety of mtDNA alterations (germline and/or somatic point mutations/deletions, mtDNA depletion and MSI) could provide rational explanation for many of the metabolic features of solid tumor biology. At the same time genetic background (mtDNA haplogroups and variations) is important in modifying the individual’s risk of cancer. Therefore, the aetiology of the protective or detrimental effect of variants/mutations should be considered in the context of mtDNA haplogroups. Moreover, the grade of heteroplasmy and the phenotypic threshold effect may play a major role in the impairment of mitochondria in pathological conditions. Finally, mtDNA is not under the stringent control of the cell cycle and intergenomic signalling pathways in carcinogenesis could be exploited in cancer therapeutics.
